# Chemical Composition of Hexane Extract of *Citrus aurantifolia* and Anti-*Mycobacterium tuberculosis* Activity of Some of Its Constituents

**DOI:** 10.3390/molecules170911173

**Published:** 2012-09-19

**Authors:** Nallely E. Sandoval-Montemayor, Abraham García, Elizabeth Elizondo-Treviño, Elvira Garza-González, Laura Alvarez, María del Rayo Camacho-Corona

**Affiliations:** 1 Facultad de Ciencias Químicas, Universidad Autónoma de Nuevo León, Av. Universidad S/N, Ciudad Universitaria, San Nicolás de los Garza CP 66451, Nuevo León, Mexico; 2 Facultad de Medicina, Universidad Autónoma de Nuevo León, Madero y Aguirre Pequeño, Mitras Centro, Monterrey CP 64460, Nuevo León, Mexico; 3 Centro de Investigaciones Químicas, Universidad Autónoma del Estado de Morelos, Av. Universidad 1001, Chamilpa, Cuernavaca CP 62209, Morelos, Mexico

**Keywords:** *Citrus aurantifolia*, *Mycobacterium tuberculosis*, multidrug resistant, coumarins, fatty acids, GC-MS analysis

## Abstract

The main aim of this study was to isolate and characterize the active compounds from the hexane extract of the fruit peels of *Citrus aurantiifolia*, which showed activity against one sensitive and three monoresistant (isoniazid, streptomycin or ethambutol) strains of *Mycobacterium tuberculosis* H_37_Rv. The active extract was fractionated by column chromatography, yielding the following major compounds: 5-geranyloxypsoralen (**1**); 5-geranyloxy-7-methoxycoumarin (**2**); 5,7-dimethoxycoumarin (**3**); 5-methoxypsoralen (**4**); and 5,8-dimethoxypsoralen (**5**). The structures of these compounds were elucidated by 1D and 2D NMR spectroscopy. In addition, GC-MS analysis of the hexane extract allowed the identification of 44 volatile compounds, being 5,7-dimethoxycoumarin (15.79%), 3-methyl-1,2-cyclopentanedione (8.27%), 1-methoxy-ciclohexene (8.0%), corylone (6.93%), palmitic acid (6.89%), 5,8-dimethoxypsoralen (6.08%), α-terpineol (5.97%), and umbelliferone (4.36%), the major constituents. Four isolated coumarins and 16 commercial compounds identified by GC-MS were tested against *M. tuberculosis* H_37_Rv and three multidrug-resistant *M. tuberculosis* strains using the Microplate Alamar Blue Assay. The constituents that showed activity against all strains were **5** (MICs = 25–50 μg/mL), **1** (MICs = 50–100 μg/mL), palmitic acid (MICs = 25–50 μg/mL), linoleic acid (MICs = 50–100 μg/mL), oleic acid (MICs = 100 μg/mL), 4-hexen-3-one (MICs = 50–100 μg/mL), and citral (MICs = 50–100 μg/mL). Compound **5** and palmitic acid were the most active ones. The antimycobacterial activity of the hexane extract of *C. aurantifolia* could be attributed to these compounds.

## 1. Introduction

With 8.9–9.9 million new and relapse cases reported every year, tuberculosis is still a matter of concern for many scientists around the World [1]. Tuberculosis is an infectious disease caused mainly by *Mycobacterium tuberculosis*, a bacterium that has developed resistance to first and second line antitubercular drugs. Efforts to treat and cure tuberculosis have been relatively unsuccessful due to the emergence of multi-drug resistant strains of *M. tuberculosis*, together with an increased incidence of new tuberculosis cases, including those associated to the Human Immunodeficiency Virus (HIV) [2]. Therefore, ongoing research is focused on the discovery of new antitubercular compounds with improved efficacy, safety, and potency. In some recent reviews, plants, marine organisms, fungi, and bacteria have all been reported as promising sources of antimycobacterial natural products, which could be considered for further drug research and development [3,4,5,6,7]. In the meantime, our research group has directed efforts to the discovery of new antimycobacterial compounds from some Mexican medicinal plants [8,9,10,11].

Mexican lime, *Citrus aurantifolia* (Christim) Swingle (Rutaceae) was recently postulated to be a hybrid between citron (a cluster of *C. medica* and *C. indica*) and *C. micrantha* by phylogenetic studies and is considered as a native species from Southeast Asia (Indo-Malayan region) [12]. *C. aurantifolia* is widespread in tropical and subtropical regions around the World such as North America (Florida, Texas, California, Mexico, *etc*.), India, Egypt, and Central America [13]. Lime essential oils are not only used as flavoring agents in beverages, manufactured foods, and pharmaceutical forms, but also as ingredients in perfumes [13]. Additionally, *C. aurantifolia* is used in traditional medicine as an antiseptic, anthelmintic, mosquito bite repellent, for stomach ailments, tonic, antiscorbutic, astringent, diuretic, headache, arthritis, digestive and appetite stimulant, and for colds, coughs and sore throats [13,14]. Previous investigations of *C. aurantifolia* have reported flavonoids, coumarins, and terpenoids [15,16,17,18]. Peel oil of *C. auranifolia* has been analyzed by GC-MS analysis several times [19,20]. Lime peel oil has shown antimicrobial [21], radical scavenging, anti-cholinesterase [22], anthelmintic [23], and anticancer activities [24]. Furthermore, leaves of lime showed protective effect against osteoporosis [25] and induced platelet aggregation [17].

Recently, our research group reported that the hexane extract of fruit peels of *C. aurantifolia* exhibited important activity against isoniazid, streptomycin or ethambutol monoresistant *M. tuberculosis* strains with minimum inhibitory concentrations (MIC) in the 25 to 50 μg/mL range [8]. Therefore, the oily extract was subjected to further chemical and antimycobacterial studies in order to identify the active compounds. 

## 2. Results and Discussion

### 2.1. Isolation and Structure Characterization of C. aurantifolia Constituents

**Figure 1:**
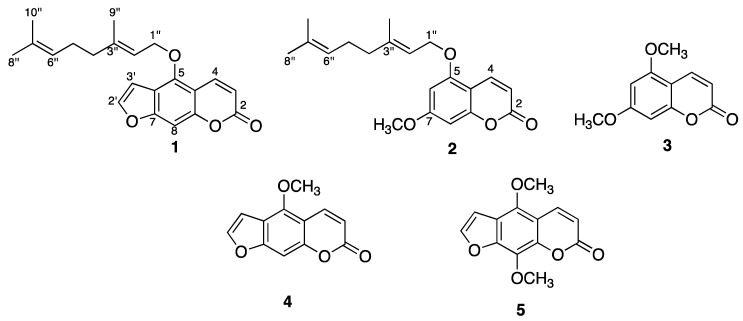
Chemical structures of isolated coumarins.

The active hexane extract was fractionated by column chromatography (CC). The fractions were subjected to several chromatographic procedures to yield compounds **1–5**. The structure of these compounds were established by 1D and 2D NMR spectra as 5-geranyloxypsoralen (bergamottin, **1**) [26], 5-geranyloxy-7-methoxycoumarin (**2**) [27], 5,7-dimethoxycoumarin (limettin, **3**) [28], 5-methoxy-psoralen (bergapten, **4**) [29], and 5,8-dimethoxypsoralen (isopimpinellin, **5**) [30] (Figure 1). It is worth mentioning that the ^1^H-NMR spectra for compounds **1** and **4** at 700 MHz exhibited long-range coupling constant values for the H4, H8, H3' protons (see Experimental section), which is a condition previously reported for polycyclic aromatics [31]. Five-bond coupling constants (^5^*J*) were observed for protons H8 (t, ^5^*J*_H4,H8_, ^5^*J*_H3',H8_ = 0.7 Hz), H4 (dd, ^5^*J*_H4,H8_ = 0.7 and ^3^*J*_H3,H4_ = 9.8 Hz), and H3' (dd, ^5^*J*_H3',H8_ = 0.7 and ^3^*J*_H3',H2'_ = 2.8 Hz) in furanocoumarin **1**. Similar long-range coupling constant patterns were observed for protons H8 (dd, ^5^*J*_H4,H8_ = 0.7 and ^5^*J*_H3',H8_ = 1.4 Hz), H4 (dd, ^5^*J*_H4,H8_ = 0.7 and ^3^*J*_H3,H4_ = 9.8 Hz), and H3' (dd, ^5^*J*_H3',H8_ = 1.4 and ^3^*J*_H3',H2'_ = 2.1 Hz) in compound **4**.

### 2.2. GC-MS Analysis

The GC-MS analysis of the hexane extract of *C. aurantifolia* fruit peel led to the identification of 44 volatile components, including monoterpenes (16.00%), sesquiterpenes (6.55%), coumarins (27.37%), fatty acids (9.78%), and some other oxygenated aromatic and non-aromatic compounds (40.30%) (Table 1). The main components were identified as 5,7-dimethoxycoumarin (**3**, 15.79%), 3-methyl-1,2-cyclopentanedione (8.27%), 1-methoxy-cyclohexene (8.0%), corylone (6.93%), palmitic acid (6.89%), 5,8-dimethoxypsoralen (**5**, 6.08%), α-terpineol (5.97%), and umbelliferone (4.36%). 

**Table 1:** Volatile constituents from the hexane extract of *C. aurantifolia*.

Peak	Compound	RT *^a^*	RI *^b^*	% *^c^*
1	Tetrahydro-2-methyl-2 *H*-pyran	5.25	838	0.72
2	4-Hexen-3-one	5.54	855	0.51
3	3-Methyl-3-penten-2-one	5.61	859	0.33
4	3-Hexen-2-one	5.67	862	0.48
5	2,3-Dimethyl-2,3-butanediol	6.30	898	1.67
6	Resorcinol	8.35	1016	3.65
7	*p-*Cymene	8.85	1046	0.36
8	1-Methoxycyclohexene	9.56	1089	8.00
9	Linalool oxide	9.97	1115	1.18
10	Crysantenile acetate	10.60	1156	0.40
11	Corylone	10.92	1177	6.93
12	Terpinen-4-ol	11.47	1213	1.66
13	α-Terpineol	11.74	1232	5.97
14	3-Methyl-1,2-cyclopentanedione	12.10	1257	8.27
15	3,7-Dimethyl-(*Z*)*-*2,6-octadienal	12.36	1276	1.09
16	Carvone	12.49	1284	0.88
17	Geraniol	12.60	1292	1.15
18	Citral	12.77	1305	2.21
19	1,8-Dimethyl-4-(1-methylethyl)-spiro[4.5]dec-8-en-7-one	12.96	1318	0.56
20	Geranyl formate	13.11	1329	0.70
21	Oleic acid	13.93	1390	0.69
22	7-Methyl-(*Z*)-8*-*tetradecen-1-ol acetate	14.20	1410	2.83
23	Geranyl acetone	14.78	1455	1.84
24	Bergamotene	14.96	1470	1.00
25	(*Z*)*-*8-Methyl-9-tetradecenoic acid	15.28	1494	1.24
26	*trans*-α-Bisabolene	15.89	1545	1.02
27	Caryophyllene oxide	17.05	1643	3.02
28	Spathulenol	17.60	1691	1.95
29	Umbelliferone	19.06	1828	4.36
30	(*Z*)*-*11(13,14-Epoxy)tetradecen-1-ol acetate	19.29	1849	0.59
31	*trans*-Phytol	19.52	1872	0.22
32	1-Heptatriacontanol	19.65	1884	0.42
33	Versalide	20.08	1926	0.51
34	Methyl palmitate	20.45	1964	0.29
35	Palmitic acid	21.19	2031	6.89
36	5,7-Dimethoxycoumarin	21.83	2083	15.80
37	5-Methoxypsoralen	22.55	2154	1.14
38	Linoleic acid	22.77	2179	0.96
39	Tricosane	23.88	2305	0.31
40	5,8-Dimethoxypsoralen	24.12	2332	6.08
41	Pentacosane	25.86	2506	0.46
42	Tetracosanal	27.80	2650	0.70
43	Octacosane	28.78	2711	0.39
44	Nonacosane	33.40	2915	0.50

*^a^* RT Retention time (min); *^b^* RI Retention index calculated for each compound; *^c^* % Relative abundances from the peak area integration.

Chemical differences in the composition of essential lime oils should be considered when an extraction process is proposed, which could account for the presence or absence of active compounds against specific diseases. Previous GC-MS chemical studies of essential oils obtained from fruit or leaves of *C. aurantifolia* have been reported to account for slight chemo-qualitative and chemo-quantitative differences, depending on which country the limes came from. The fruit peel essential oil of Iranian *C. aurantifolia* was reported to contain 50 compounds, having limonene (53.53%), α-terpineol (9.41%), and γ-terpinene (6.26%) as the most abundant [21]. Distilled oil of fruit peels of *C. aurantifolia* from southern Florida was studied by GC-MS detecting between 50 to 60 volatiles with limonene (32.6%), α-terpineol (12.5%), and β-pinene (6.3%) as the main compounds [20]. On the other hand, an Australian research group reported the analysis of the essential oil of Mexican peel lime with limonene (30.5%) and γ-terpinene (19.2%) as the main components, together with some minor constituents as geranial (5.9%), 7-methoxycoumarin (3.3%), 5,7-dimethoxycoumarin (6.6%), and bergapten (2.9%) [32]. These reports showed that limonene and α-terpineol are the most common and abundant components of the essential oil of *C. aurantifolia*. 

A chemical reinvestigation of Mexican lime oil of *C. aurantifolia* led to the identification of 98 compounds, suggesting that during distillation, lime oil undergoes modifications in its chemical composition because heating of the juice-oil emulsion and an acidic environment provoke transformations that generate more stable compounds. Authors provided insights into the acid catalyzed hydrolysis and rearrangement reactions of the bicyclic hydrocarbons named α- and β-terpinene, sabinene, and α-thujene, which generate alcohols (α-terpineol, terpinen-4-ol, *endo*-fenchol, borneol, isoborneol) and hydrocarbons (terpinolene, limonene, fenchene, camphene, γ-terpinene, α-terpinene) [33]. These insights could explain at some point the chemo-qualitative and chemo-quantitative differences from one sample to another. It is worth mention that the extraction processes of oils accounted for those chemical differences. Chemo-qualitative analysis of the volatile components exhibited substantial differences between the hexane extract of this research and the distilled oil of *C. aurantifolia* of previous reports, detecting 5,7-dimethoxycoumarin, 5,8-dimethoxypsoralen, and α-terpineol as the most abundant constituents, in contrast to limonene and α-terpineol of the distilled oil. Previous studies of essential lime oils reported limonene as the most abundant component, but in this work limonene is found in trace amounts in the hexane extract. 

### 2.3. Antimycobacterial Activity of Constituents from C. aurantifolia

Four isolated compounds and sixteen commercial substances identified by GC-MS were evaluated against sensitive and multidrug-resistant *M. tuberculosis* strains. The molecules that showed activity against all strains were 5,8-dimethoxypsoralen (**5**, MICs = 25–50 μg/mL), 5-geranyloxypsoralen (**1**, MICs = 50−100 μg/mL), palmitic acid (MICs = 25−50 μg/mL), linoleic acid (MICs = 50−100 μg/mL), oleic acid (MICs = 100 μg/mL), 4-hexen-3-one (MICs = 50−100 μg/mL), and citral (MICs = 50 μg/mL). All tested compounds exhibited less antimycobacterial activity than the positive controls used, ethambutol, isoniazid and rifampicin (Table 2). 

Biological results showed that 5,8-dimethoxypsoralen (**5**) inhibited cellular growth of multidrug-resistant *M. tuberculosis* strains with MIC values in the range of 25–50 μg/mL. Analysis of the structure-activity relationship between coumarins and furanocoumarins indicates that furan moiety contributes to potency, as can be observed for furanocoumarins **5** (MICs = 25 and 50 μg/mL) and **1** (MICs = 50 and 100 μg/mL) in comparison to coumarins **2** and **3** (MICs > 200 μg/mL) (Table 2). The possible mechanism of action could be related to previous reports in which certain furanocoumarins can not only intercalate into deoxyribonucleic acid (DNA) and create cross-links with thymidine residues, but also can form covalent links to apoproteins and permanently inactivate cytochrome P450 enzymes [34]. It has been reported in the literature that compound **5** possesses antimicrobial [35], spasmogenic [36], cardiovascular [37], cancer chemopreventive [38], vasorelaxing [39], and non-phototoxic effects [40,41]. Additionally, compound **1** possesses antimutagenic effects [42] and it is a cancer chemopreventive agent [43]. 

**Table 2:** MIC values (μg/mL) of constituents from *C. aurantifolia* against *M. tuberculosis*.

Compound	H37Rv *^a^*	H10 *^b^*	M15 *^c^*	M26 *^d^*
5-Geranyloxypsoralen (**1**)	50	200	100	100
5-Geranyloxy-7-methoxycoumarin (**2**)	200	200	100	100
5,7-Dimethoxycoumarin (**3**)	>200	NT	NT	NT
5,8-Dimethoxypsoralen (**5**)	25	25	>50	50
4-Hexen-3-one	50	>200	>200	>200
3-Methyl-3-penten-2-one	>200	NT	NT	NT
Resorcinol	>200	NT	NT	NT
*p-*Cymene	>200	NT	NT	NT
Linalool oxide	>200	NT	NT	NT
Terpinen-4-ol	200	>200	>200	>200
3-Methyl-1,2-cyclopentanedione	>200	NT	NT	NT
Carvone	200	>200	>200	>200
Geraniol	200	>200	>200	>200
Citral	50	>200	>200	200
Geranyl formate	>200	NT	NT	NT
Oleic acid	100	100	100	100
Methyl palmitate	>200	NT	NT	NT
Palmitic acid	25	50	50	50
Linoleic acid	50	100	100	100
Pinacol	>200	NT	NT	NT
Ethambutol	2.0	15	15	15
Isoniazid	0.02	5	7	6
Rifampicin	0.08	9	10	12

*^a^ M. tuberculosis* H_37_Rv strain is sensitive to isoniazid, rifampicin, streptomycin and ethambutol; *^b,c,d^ M. tuberculosis* H10, M15 and M26 are clinical strains resistant to isoniazid and rifampicin; NT: Not tested.

The saturated fatty acid palmitic acid exhibited higher activity against multidrug-resistant *M. tuberculosis* strains (MICs = 50 μg/mL) than the unsaturated fatty acids oleic acid and linoleic acid, which showed less activity (MICs = 100 μg/mL). Saravanakumar’s research group reported the activity of oleic acid (MIC 25 µg/mL), linoleic acid (MIC 50 µg/mL), and palmitic acid (no significant) against *M. tuberculosis* H37Rv using the Bactec-460 method [44]. For both studies, there was agreement for linoleic acid (MIC 50 µg/mL). However, in this study, the results for oleic acid (MIC 100 µg/mL) and palmitic acid (MIC 25 µg/mL) against H37Rv using the Microplate Alamar Blue Assay were the opposite of those reported by Saravanakumar. On the other hand, Hirsch and Barchet published the bacteriostatic activity of saturated fatty acids C_10_–C_16_ against *M. tuberculosis* and those results are in agreement with the antimycobacterial activity of palmitic acid (C16:0) found in our study [45]. Reports in the literature have shown that long-chain unsaturated fatty acids such as oleic and linoleic acid are selective inhibitors of the enoyl-acyl carrier protein reductase (FabI), which account for their antibacterial activities through the inhibition of fatty acid synthesis [46]. Therefore, these experimental findings could be considered to explain the antimycobacterial activities of oleic and linoleic acids. 

## 3. Experimental

### 3.1. General Experimental Procedures

1D and 2D NMR spectra were recorded on Varian spectrometers at 400 and 700 MHz using CDCl_3_ as solvent and TMS as the internal standard. CC was carried out to fractionate the active extract. Fractions were monitored by Si gel thin layer chromatography and observed under UV light at 254 and 364 nm. The analysis of volatile constituents of hexane extract was performed on a HP Agilent Technologies 6890 gas chromatograph equipped with a MSD 5973 quadrupole mass detector (HP Agilent, CA, USA) in electron impact mode at 70 eV. On the other hand, *in vitro* anti-tuberculosis test was performed using the Alamar Blue microassay.

### 3.2. Chemicals

Silica gel (70–230 mesh, Merck®). Precoated silica gel Al foils (Fluka®). All solvents used were analytical grade (CTR México). C_7_-C_40_ n-alkanes, dimethyl sulphoxide (DMSO), glycerol, Tween 80, 4-hexen-3-one, 3-methyl-3-penten-2-one, resorcinol, *p*-cymene, linalool oxide, terpinen-4-ol, 3-methyl-1,2-cyclopentanedione, carvone, geraniol, citral, geranyl formate, oleic acid, methyl palmitate, palmitic acid, linoleic acid, pinacol, ethambutol, isoniazid, rifampicin were purchased from Sigma-Aldrich (St. Louis, MO, USA). Middlebrook 7H9 broth and OADC (oleic acid-album-dextrosa-catalasa) from Becton Dickinson Co. (Franklin Lakes, NJ, USA), and Alamar blue solution from Trek Diagnostic (Westlake, OH, USA).

### 3.3. Mycobacterium Tuberculosis Strains

*M. tuberculosis* H_37_Rv (27294) sensitive to all five first-line antituberculosis drugs (streptomycin, isoniazid, rifampicin, ethambutol and pirazinamide) was obtained from the American Type Culture Collection (ATCC). Multidrug resistant *M. tuberculosis* H10, M15, and M26 strains were obtained from sputum of tuberculosis patients. These specimens were kindly provided by Dr. Virgilio Bocanegra-García from the Centro de Biotecnología Genomica del Instituto Politécnico Nacional. The local ethics committee approved all protocols used in this study.

### 3.4. Plant Material

Fruits and flowering branches of *Citrus aurantifolia* were collected in Montemorelos, Nuevo León, México in May 2009. A voucher specimen (Number: 024769) was deposited at the Herbarium of the Faculty of Biological Sciences of the Autonomous University of Nuevo León. 

### 3.5. Extraction and Isolation of Constituents from C. aurantifolia

Peels (1.4 kg) were removed from fresh fruits (7.8 kg) and macerated twice with *n*-hexane (6 L) for 72 h at room temperature. Solvent was removed under reduced pressure to give a yellowish oily residue (16.05 g). The *n*-hexane extract (16.00 g) was subjected to silica gel CC with gradient elution of hexane–EtOAc affording 24 fractions (F1 100:0; F2 95:5; F3-7 90:10; F8-13 85:15; F14-16 80:20; F17-18 75:25; F19 70:30; F20 60:40; F21 50:50; F22 40:60; F23 30:70-10:90; F24 0:100 hex/EtOAc). Fraction 3 (640 mg) eluted with hexane–EtOAc (90:10) was subjected to silica gel CC with gradient elution as above. Sub-fractions eluted with 98:2 hexane–EtOAc led to the purification of 24.8 mg of **1** as a yellowish oily compound. Fraction 5 (1.16 g) eluted with hexane–EtOAc (90:10) afforded a white solid, which was recrystallized from hexane–EtOAc (97:3) to give 17.6 mg of **2**. Fractions 9–10 (150 mg) obtained from hexane–EtOAc (85:15) gave a white precipitate which was recrystallized in chloroform to give 10.7 mg of **3** as colorless needles. Fractions 11–12 (440 mg) obtained from hexane–EtOAc (85:15) were similarly subjected to silica gel CC with gradient elution using hexane–EtOAc. Sub-fractions 61–120 (hexane–EtOAc, 96:4) afforded 56.2 mg of **4** as a yellow solid. Fractions 13–15 (560 mg) eluted with hexane–EtOAc (80:20) were chromatographed as above. Sub-fractions eluted with 95:5 hexane–EtOAc gave 4 mg of **5** as a yellow solid.

### 3.6. Spectroscopical Data

*5-Geranyloxypsoralen* (**1**). ^1^H-NMR (700 MHz, CDCl_3_) δ: 8.17 (1H, dd, *J* = 9.8, 0.7 Hz, H-4), 7.59 (1H, d, *J* = 2.8 Hz, H-2'), 7.16 (1H, t, *J* = 0.7 Hz, H-8), 6.96 (1H, dd, *J* = 2.8, 0.7 Hz, H-3'), 6.27 (1H, d, *J* = 9.8 Hz, H-3), 5.53 (1H, tq, *J* = 6.3, 1.4 Hz, H-2"), 5.06 (1H, m, H-6"), 4.95 (2H, d, *J* = 6.3 Hz, H-1"), 2.09 (4H, m, H-5" y H-4"), 1.69 (3H, d, *J* = 0.7 Hz, H-9"), 1.68 (3H, d, *J* = 0.7 Hz, H-8"), 1.60 (3H, d, *J* = 0.7 Hz, H-10"). ^13^C-NMR (176 MHz, CDCl_3_) δ: 161.57 (C-2), 158.34 (C-7), 152.86 (C-8a), 149.5 (C-5), 145.18 (C-2'), 143.27 (C-3"), 139.86 (C-4), 132.27 (C-7"), 123.69 (C-6"), 119.05 (C-2"), 114.27 (C-6), 112.77 (C-3), 107.73 (C-4a), 105.28 (C-3'), 94.45 (C-8), 69.95 (C-1"), 39.71 (C-4"), 26.40 (C-5"), 25.90 (C-8"), 17.94 (C-9"), 16.90 (C-10"). 

*5,7-Dimethoxycoumarin* (**3**). ^1^H-NMR (400 MHz, CDCl_3_) δ: 7.97 (1H, d, *J* = 9.6 Hz, H-4), 6.42 (1H, d, *J* = 2 Hz, H-8), 6.28 (1H, d, *J* = 2.4 Hz, H-6), 6.16 (1H, d, *J* = 9.6 Hz, H-3), 3.89 (3H, s, 7-OCH_3_), 3.85 (3H, s, 5-OCH_3_). ^13^C-NMR (100 MHz, CDCl_3_) δ: 163.65 (C-2), 161.57 (C-7), 156.92 (C-5), 156.76 (C-10), 138.77 (C-4), 110.89 (C-3), 103.96 (C-9), 94.80 (C-6), 92.72 (C-8), 55.91 (CH_3_O-7), 55.78 (CH_3_O-5).

*5-Methoxypsoralen* (**4**). ^1^H-NMR (700 MHz, CDCl_3_) δ: 8.16 (1H, dd, *J* = 9.8, 0.7 Hz, H-4), 7.6 (1H, d, *J* = 2.8 Hz, H-2'), 7.14 (1H, dd, *J* = 1.4, 0.7 Hz, H-8), 7.02 (1H, dd, *J* = 2.1, 1.4 Hz, H-3'), 6.28 (1H, d, *J* = 9.8 Hz, H-3), 4.27 (3H, s, 5-OCH_3_). ^13^C-NMR (176 MHz, CDCl_3_) δ: 161.53 (C-2), 158.59 (C-7), 152.90 (C-4a), 149.78 (C-5), 145.01 (C-2'), 139.53 (C-4), 112.87 (C-6), 112.76 (C-3), 106.61 (C-8a), 105.26 (C-3'), 94.07 (C-8), 60.30 (5-OCH_3_). 

### 3.7. GC-MS Analysis

Chemical composition of volatile compounds from the active hexane extract was analysed on a gas chromatograph equipped with a quadrupole mass detector in electron impact mode at 70 eV. Volatile compounds were separated on a HP 5MS capillary column (25 m long, 0.2 mm i.d., 0.3 μm film thickness). The oven temperature was set at 40 °C for 2 min and then programmed from 40 to 260 °C at 10 °C/min, and keep it 20 min at 260 °C. Mass detector conditions were as follows: interphase temperature 200 °C and mass acquisition range 20–550. Temperature of injector and detector were set to 250 °C and 280 °C, respectively. The splitless injection mode was carried out with 1 μL of oily extract. The carrier gas was helium at a flow rate of 1 mL/min. Identification of volatiles was performed comparing their mass spectra with those of the National Institute of Standards and Technology NIST 1.7 library. In addition, a standard solution of C7-C40-alkanes was used to obtain the retention index of compounds and comparing them with literature data [47]. Semi-quantitative data were calculated from the GC peak areas without using correction factors and were expressed as relative percentage (peak area %) of the total volatile constituents identified.

### 3.8. Antimycobacterial Activity

The activity of all compounds against the *M. tuberculosis* strains was determined using the Microplate Alamar Blue Assay (MABA) as previously described in the literature [8,9].

## 4. Conclusions

5,8-Dimethoxypsoralen (**5**, MICs = 25−50 μg/mL), 5-geranyloxypsoralen (**1**, MICs = 50−100 μg/mL), palmitic acid (MICs = 25−50 μg/mL), linoleic acid (MICs = 50−100 μg/mL), oleic acid (MICs = 100 μg/mL), 4-hexen-3-one (MICs = 50−100 μg/mL), and citral (MICs = 50 μg/mL) are responsible for the antimycobacterial activity observed in *C. aurantifolia*. Compound **5** and palmitic acid were the most active constituents. Utilization of these compounds for therapeutic purposes will require the evaluation of their cytotoxic activities and determination of their index values. 

## References

[1] World Health Organization Home Page http://www.who.int/tb/publications/2011/factsheet_tb_2011.pdf.

[2] Tripathi R.P., Tewari N., Dwivedi N., Tiwari V.K. (2005). Fighting tuberculosis: An old disease with new challenges. Med. Res. Rev..

[3] Copp B.R. (2003). Antimycobacterial natural products. Nat. Prod. Rep..

[4] Okunade A.L., Elvin-Lewis M.P.F., Lewis W.H. (2004). Natural antimycobacterial metabolites: Current status. Phytochemistry.

[5] Nayyar A., Jain R. (2005). Recent advances in new structural classes of anti-tuberculosis agents. Curr. Med. Chem..

[6] Copp B.R., Pearce A.N. (2007). Natural product growth inhibitors of *Mycobacterium tuberculosis*. Nat. Prod. Rep..

[7] García A., Bocanegra-García V., Palma-Nicolás J.P., Rivera G. (2012). Recent advances in antitubercular natural products. Eur. J. Med. Chem..

[8] Camacho-Corona M.R., Ramírez-Cabrera M.A., González-Santiago O., Garza-González E., Palacios I.P., Luna-Herrera J. (2008). Activity against drug resistant-tuberculosis strains of plants used in Mexican traditional medicine to treat tuberculosis and other respiratory diseases. Phytother. Res..

[9] Camacho-Corona M.R., Favela-Hernández J.M.J., González-Santiago O., Garza-González E., Molina-Salinas G.M., Said-Fernández S., Delgado G., Luna-Herrera J. (2009). Evaluation of some plant-derived secondary metabolites against sensitive and multidrug-resistant *Mycobacterium tuberculosis*. J. Mex. Chem. Soc..

[10] Favela-Hernández J.M.J., García A., Garza-González E., Rivas-Galindo V.M.,  Camacho-Corona M.R. (2012). Antibacterial and antimycobacterial lignans and flavonoids from *Larrea tridentata*. Phytother. Res..

[11] Esquivel-Ferriño P.C., Favela-Hernández J.M., Garza-González E., Waksman N., Ríos M.Y., Camacho-Corona M.R. (2012). Antimycobacterial activity of constituents from *Foeniculum vulgare* var. dulce grown in México. Molecules.

[12] Nicolosi E., Deng Z.N., Gentile A., La Malfa S., Continella G., Tribulato E. (2000). *Citrus* phylogeny and genetic origin of important species as investigated by molecular markers. Theor. Appl. Genet..

[13] Morton J. (1987). Mexican Lime. Fruits of Warm Climates.

[14] Apraj V., Thakur N.D., Bhagwat A., Mallya R., Sawant L., Pandita N. (2011). Pharmacognostic and phytochemical evaluation of *Citrus aurantifolia* (Christm) Swingle peel. Pharmacogn. J..

[15] Feger W., Brandauer H., Ziegler H. (2000). Sesquiterpene hydrocarbons of cold-pressed lime oils. Flav. Frag. J..

[16] Johann S., Smania A., Pizzolatti M.G., Schripsema J., Braz-Filho R., Branco A. (2007). Complete ^1^H and ^13^C-NMR assignments and antifungal activity of two 8-hydroxy flavonoids in mixture. An. Acad. Bras. Cienc..

[17] Piccinelli A.L., Garcia M.M., Armenteros D.M., Alfonso M.A., Arevalo A.C., Campone L., Rastrelli L. (2008). HPLC-PDA-MS and NMR characterization of C-glycosyl flavones in a hydroalcoholic extract of *Citrus aurantifolia* leaves with antiplatelet activity. J. Agric. Food. Chem..

[18] Jiwajinda S., Santisopasri V., Ohigashi H. (2000). Coumarin-related compounds as plant growth inhibitors from two rutaceous plants in thailand. Biosci. Biotechnol. Biochem..

[19] Afolayan A.J., Asekun O.T. (2008). Comparative study of the chemical profiles of the essential oils of ripe and rotten fruits of *Citrus aurantifolia* Swingle. Nat. Prod. Commun..

[20] Chisholm G.M., Wilson M.A., Gaskey G.M. (2003). Characterization of aroma volatiles in the key lime essential oils (*Citrus aurantifolia* Swingle). Flav. Frag. J..

[21] Jafari S., Esfahani S., Fazeli M.R., Jamalifar H., Samadi M., Samadi N., Najarian-Toosi A., Shams-Ardekani M.R., Khanavi M. (2011). Antimicrobial activity of lime essential oil against food-borne pathogens isolated from cream-filled cakes and pastries. Int. J. Biol. Chem..

[22] Tundis R., Loizzo M.R., Bonesi M., Menichini F., Mastellone V., Colica C., Menichini F. (2012). Comparative study on the antioxidant capacity and cholinesterase inhibitory activity of *Citrus aurantifolia* Swingle, *C. aurantium* L., and *C. bergamia* Risso and Poit. peel essential oils. J. Food. Sci..

[23] Taur D.J., Kulkarni V.B., Patil R.Y., Patil R.N. (2009). Anthelmintic activity of *Ocimum sanctum* and *Citrus aurantifolia* oils. Pharmacologyonline.

[24] Gharagozloo M., Doroudchi M., Ghaderi A. (2002). Effects of *Citrus aurantifolia* concentrated extract on the spontaneous proliferation of MDA-MB-453 and RPMI-8866 tumor cell lines. Phytomedicine.

[25] Shalaby N.M.M., Abd-Alla H.I., Ahmed H.H., Basoudan N. (2011). Protective effect of *Citrus*
*sinensis* and *Citrus aurantifolia* against osteoporosis and their phytochemical constituents. J. Med. Plants Res..

[26] Kawaii S., Tomono Y., Katase E., Ogawa K., Yano M. (1999). Isolation of furanocoumarins from bergamot fruits as HL-60 differentiation-inducing compounds. J. Agric. Food Chem..

[27] Miyake Y., Hiramitsu M. (2011). Isolation and extraction of antimicrobial substances against oral bacteria from lemon peel. J. Food Sci. Technol..

[28] Shiota H. (1990). Volatile components in the peel oil from fingered citron (*Citrus medica* L. var. *sarcodactylis* Swingle). Flav. Frag. J..

[29] Hongwei Y., Bogang L., Changsong L., Guolin Z. (2010). Chemical Study on *Evodia vestita*. Chin. J. Appl. Environ. Biol..

[30] Miyazawa M., Tsukamoto T., Anzai J., Ishikawa Y. (2004). Insecticidal effect of phthalides and furanocoumarins from *Angelica acutiloba* against *Drosophila melanogaster*. J. Agric. Food Chem..

[31] Evans F.E., Fu P.P., Cairns T. (1981). Long-range coupling constants for structural analysis of complex polycyclic aromatic hydrocarbons by high-field proton magnetic resonance spectrometry. Anal. Chem..

[32] Craske J.D., Suryadi N., Wootton M.A. (2005). Comparison of the peel oil components of Australian native lime (*Microcitrus australe*) and Mexican lime (*C. aurantifolia* Swingle). J. Sci. Food Agric..

[33] Chamblee T.S., Clark B.C. (1997). Analysis and chemistry of distilled lime oil (*Citrus aurantifolia* Swingle). J. Essent. Oil Res..

[34] Bourgaud E., Hehn A., Larbat R., Doerper S., Gontier E., Kellner S., Matern U. (2006). Biosynthesis of coumarins in plants: A major pathway still to be unraveled for cytochrome P450 enzymes. Phytochem. Rev..

[35] Ngwendson J.N., Bedir E., Efange S.M., Okunji C.O., Iwu M.M., Schuster B.G., Khan I.A. (2003). Constituents of *Peucedanum zenkeri* seeds and their antimicrobial effects. Pharmazie.

[36] Abbaskhan A., Choudhary M.I., Ghayur M.N., Parween Z., Shaheen F., Gilani A.U., Maruyama T., Iqbal K., Tsuda Y. (2012). Biological activities of Indian celery, *Seseli diffusum* (Roxb. Ex Sm.) Sant. & Wagh. Phytother. Res..

[37] Guo S., Li S., Peng Z., Ren X. (1998). Isolation and identification of active constituents of *Toddalia asiatica* in cardiovascular system. Zhong Yao Cai.

[38] Kleiner H.E., Vulimiri S.V., Starost M.F., Reed M.J., DiGiovanni J. (2002). Oral administration of the citrus coumarin, isopimpinellin, block DNA adduct formation and skin tumor initiation by 7,12-dimethylbenz(a)anthracene in SECAR mice. Carcinogenesis.

[39] Chiou W.F., Huang Y.L., Chen C.F., Chen C.C. (2001). Vasorelaxing effect of coumarins from *Cnidium monnieri* on rabbit corpus cavernosum. Planta Med..

[40] Hudson J.B., Miki N., Towers G.H. (1987). Isopimpinellin is not phototoxic to viruses and cells. Planta Med..

[41] Ivie G.W., Beier R.C. (1996). Isopimpinellin is not phototoxic in a chick skin assay. Photochem. Photobiol..

[42] Olguín-Reyes S., Camacho-Carranza R., Hernández-Ojeda S., Elinos-Baez M., Espinosa-Aguirre J.J. (2012). Bergamottin is a competitive inhibitor of CYP1A1 and is antimutagenic in the Ames test. Food Chem. Toxicol..

[43] Miyake Y., Murakami A., Sugiyama Y., Isobe M., Koshimizu K., Ohigashi H. (1999). Identification of coumarins from lemon fruit (*Citrus limon*) as inhibitors of *in vitro* tumor promotion and superoxide and nitric oxide generation. J. Agric. Food Chem..

[44] Saravanakumar D.E.M., Folb P.I., Campbell B.W., Smith P. (2008). Antimycobacterial activity of the red alga *Polysiphonia virgata*. Pharm. Biol..

[45] Hirsch I.W., Barchet H.M. (1955). Significance of medium lengh fatty acids and their salts in the prophylaxis of tuberculosis. Deutsche Zeitschrift fuer Verdauung_und Stoffwechselkrankheiten..

[46] Zhing C.J., Yoo J.S., Lee T.G., Cho H.Y., Kim Y.H., Kim W.G. (2005). Fatty acid synthesis is a target for antibacterial activity of unsaturated fatty acids. FEBS Lett..

[47] Adams R.P. (2007). Identification of Essential Oils Components by Gas Chromatography/Mass Spectrometry.

